# A clinical study of C arm-guided selective spinal nerve block combined with low-temperature plasma radiofrequency ablation of dorsal root ganglion in the treatment of zoster-related neuralgia

**DOI:** 10.3389/fneur.2023.1122538

**Published:** 2023-02-24

**Authors:** Zhen-Wu Zhang, Yan Zhao, Tian-Yi Du, Juan Zhang, Qiong Wu, Zhe-Yin Wang

**Affiliations:** Department of Pain Medicine, Shenzhen People's Hospital (The Second Clinical Medical College, Jinan University, The First Affiliated Hospital, Southern University of Science and Technology), Shenzhen, Guangdong, China

**Keywords:** zoster-related neuralgia, selective spinal nerve block, analgesic efficacy, psychological response, low-temperature plasma radiofrequency ablation

## Abstract

**Background:**

This study evaluated the analgesic efficacy and psychological response of low-temperature plasma ablation of dorsal root ganglion (DRG) combined with selective spinal nerve block in patients with acute or subacute zoster-related neuralgia (ZRN).

**Methods:**

Totally 90 ZRN patients were randomly and evenly divided into three groups. Treatment was given to Group A using C arm-guided selective spinal nerve block (C-SSVB), Group B using C-SSVB and pulsed radiofrequency (PRF), and Group C using C-SSVB and low-temperature plasma ablation of the DRG. The outcomes were examined using the Visual Analog Scale (VAS). Anxiety and depression of patients were evaluated using the Self-rating Anxiety Scale (SAS) and Self-rating Depression Scale (SDS). Quality of life was assessed using the Pittsburgh Sleep Quality Index (PSQI) and postoperative Satisfaction scale. In addition, data on adverse events and medication usage rates were collected.

**Results:**

The 90 patients were eligible for this study. The three treatments reduced VAS scores with no significant difference between groups A and B at the same time points; however, group B tended to have numerically lower VAS scores. Comparatively, group C had significantly reduced VAS scores on day 1 and 1 month after treatment compared with the other two groups. In terms of the decreasing SAS, SDS and PSQI scores, all the three treatments improved the anxiety, depression and sleep quality of the patients. In addition, significant alleviation in anxiety was found in group C compared with group A at all- time points. However, there was no statistically significant difference among the three groups in treatment-related adverse events that mainly focused on puncture pain at the surgical-site, skin numbness and medication usage rates.

**Conclusions:**

C-SSVB and LTPRA of DRG will be considered as a promising treatment option for ZRN patients if those results can be confirmed after further validation.

## Introduction

Herpes zoster (HZ) is caused by the reactivation of the varicella-zoster virus (VZV), which lies dormant in the ganglia (1). In addition to developing a the rash, HZ can inflict damage to peripheral nerves during flare-ups (2). Acute severe pain and postherpetic neuralgia (PHN) are frequent complications of HZ (3). Around 21,400 people in Switzerland are estimated to be afflicted by HZ each year, with 10–20% of those further progressing to PHN as a common long-term complication (4). The neuron destruction and inflammation caused by the VZV are often accompanied by persistent burning or intermittent needle-like pain in clinical practice, as well as a loss of vision and facial scarring, all of which lead to serious sleep disorders and lower the patient's quality of life (5, 6). Therefore, early and timely treatment of herpetic neuralgia is crucial to avoid its progression to PHN.

At present, there are no effective therapies that can target acute or subacute herpetic neuralgia. Herpetic neuralgia typically develops in less than a month, and early antiviral treatment and pain relief drugs are the common treatment approaches in clinical practice (7). Pain relief drugs such as gabapentin (GBP) or pregabalin (PGB) are most commonly used in the local treatment of zoster-related neuralgia (ZRN) (8, 9), followed by opioids if necessary (10). Unfortunately, randomized controlled trials have shown that pain severity is unsatisfactorily reduced in 20–40% of patients treated with antivirals or other drugs (11). Therefore, more effective and safer treatments are required to improve the outcomes of the disease and the quality of life of the patients.

Low-temperature plasma radiofrequency ablation (LTPRA), a novel treatment method, is shown to effectively destroy the dorsal root ganglion (DRG) through a bipolar plasma cutter head with a diameter of 1 mm in order to achieve the effects of blocking pain signaling transmission (12). Generally, the sites of predilection for HZ infections are the chest, back and abdomen. In HZ patients, the VZV may exist in the DRG in a latent state before the initial infection of the human body. DRG neurons are a diverse and complex group of cells that play a key role in the development and maintenance of neuropathic pain as well as in the transmission of nociceptive signals in addition to proprioception, temperature, and mechanical stimuli (13). DRG has thus become a research focus and is regarded as a novel therapeutic target. LTPRA, which is responsible for the dissociation of intercellular bonds in tissues based on molecular dissociation, may be an innovative approach to the treatment of neuralgia (14). This approach may offer hope to patients with neuropathic pain. The efficacy of LTPRA in relieving refractory cluster headache (15), post-amputation pain (16), trigeminal neuralgia (17), and pain caused by cervical herniation (18) has been reported so far. However, literature on low-temperature plasma ablation in the treatment of neuropathic pain is limited. Therefore, this study aimed to compare the clinical efficacies and outcomes of LTPRA in ZRN patients.

## Research objects and methods

### Research objects

The need for written consent from patients was waived because we ensured all the information and treatment records of the patients were kept anonymous by all researchers involved. Patients diagnosed with ZRN (course of disease < 1 month) with a clear history of zoster and hospitalized at the Guangdong Provincial Shenzhen People's Hospital (Ethics No. LL-KY-2022144-01) from May 2019 to December 2021 were included in this study. The inclusion criteria were: (1) patients diagnosed with ZRN with a clear history of zoster; (2) patients aged between 50 to 75 years; (3) patients with pain located in the T3-T12 spinal nerve distribution area; (4) patients with visual analogous scale (VAS) score ≥ 5, and; (5) ZRN patients only received medication 1 week prior to the study. The exclusion criteria were: (1) patients with a history of cancer, infection in the spinal canal or diabetes; (2) patients with systemic immune disease, impaired cardiac and pulmonary function or respiratory tract infection; (3) patients with presence of intercostal neuralgia but not caused by HZ, and; (4) patients with pain located beyond T3-T12 spinal nerve distribution area. In all, 90 patients were randomly allocated to group A, group B and group C, with 30 ones in each group.

### Treatment methods

**Figure 1:**
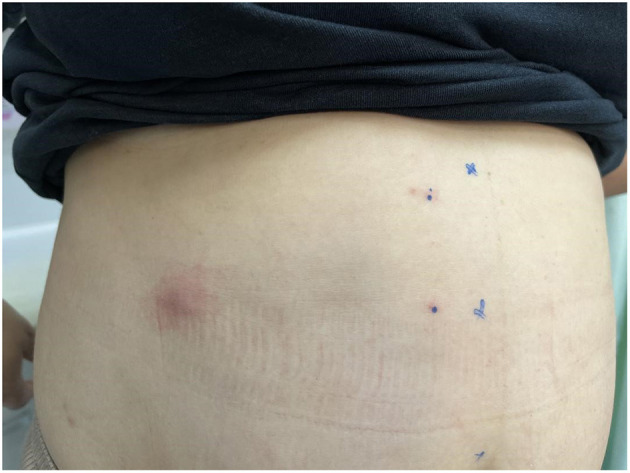
Abdominal illustration of ZRN patient for locating the target nerve based on the location of new rashes.

**Figure 2:**
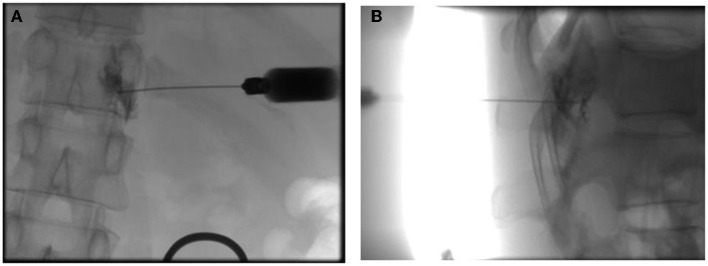
Confirmation of the extent of the block with contrast under the C-arm X-ray machine positioning. **(A)** Anteroposterior film showing the needle tip's position at the connecting line of the intervertebral foramen on the affected side and the entry of the contrast agent into the spinal canal along the nerve root. **(B)** Lateral film of the upper half of the intervertebral foramen on the affected side.

The first group, indicated as A, was treated with the C-SSVB method. Briefly, all patients underwent chest computed tomography (CT) before the treatment. Abdominal illustration of ZRN patient for locating the target nerve based on the location of new rashes was created (Figure 1). Firstly, a machine with C-arm was used to guide the thoracic spinal nerve block. The blocking solution (0.5 ml of 1% ropivacaine, 1 ml of 2% lidocaine, 1 ml of mecobalamin injection, 20 mg of triamcinolone acetonide and 2 ml of 0.9% sodium chloride) was prepared. Later, patients were placed in the prone position on the operating table with a comfortable pillow under their chest. Then, the peripheral venous access was opened, and vital life signs of the patients were monitored. Routine disinfection and draping were performed, and local anesthesia was administered. The coordinates of the needle entry point were defined at 5–9 cm next to the posterior median line of the affected area. The action of puncture with a long needle under the guidance of the C-arm was performed, and then the needle insertion angle and depth were dynamically adjusted. Puncturing was carefully performed to avoid the contusion of dural sac and nerve root. The anteroposterior film at the needle tip was placed at the connecting line of the intervertebral foramen on the affected side (Figure 2A); the lateral film was placed on the upper half of the intervertebral foramen on the affected side (Figure 2B). Then, 0.5 ml iopamidol was injected to confirm that the contrast agent had entered the spinal canal along the nerve root (Figure 2A). After the liquid drew back was void of blood and gas, 1 mL blocking solution was injected. The vital signs (including blood pressure, respiration, pulse, body temperature, and consciousness) were closely monitored during and following the operation. The treatment was repeated for different neural targets as described above.

**Figure 3:**
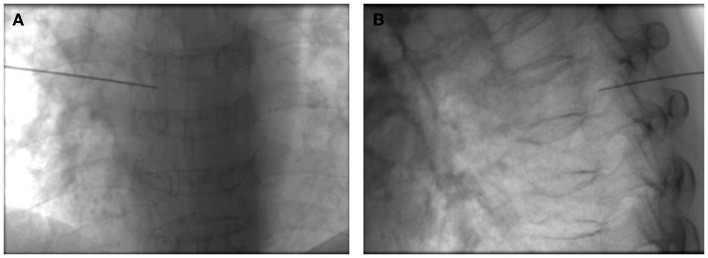
PRF treatment based on the C-SSVB treatment. Confirmation of the position of the RF target to the dorsal root ganglion in the **(A)** front and **(B)** side position of the spinal cord.

Group B was given pulse repetition frequency (PRF) treatment based on the C-SSVB treatment. Briefly, the patients were sent to the interventional operating room and transferred on the surgical bed, then the peripheral venous access was opened, and their vital signs were monitored (Figures 3A, B). The PRF was conducted using a radio frequency instrument (R-2000B, Beiqi Medical Technology Co., Ltd.). According to group A, a radiofrequency trocar (10 or 15 cm) was used to puncture the nerve target radiofrequency electrode, and 1 ml of blocking solution was injected. Electrical stimulation at 50 Hz and 0.5 V was set to induce paresthesia in the original pain area, then the electrical stimulation was set at 2 Hz and 0.5–1 V to induce muscle twitching in the original pain area, followed by treatment using pulsed radiofrequency. The treatment parameters were set as a pulse electric current at 20 ms and a voltage of 45 V at 42°C for 120 s and 5 working cycles. The treatment was repeated for different neural targets as described above.

**Figure 4:**
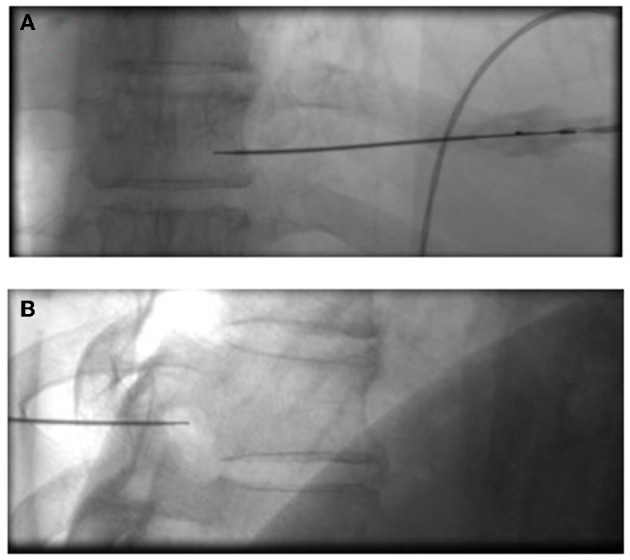
Insertion and position of the plasma knife. In the positive **(A)** and lateral position **(B)**, confirm that the target point of the plasma knife head reaches the dorsal root ganglia.

Group C was treated using LTPRA based on the C-SSVB treatment. Likewise, the patients were instructed to lay on the bed as mentioned above, the peripheral venous access was opened, and their vital signs were monitored. LTPRA was conducted using the ArthroCare SystemR-12000 (19). Under the guidance of the C-arm, the matching puncture needle was inserted into the foramen of the DRG at the corresponding stage, and a 1 ml of blocking solution was injected. The plasma knife head was inserted, and the device mode was set to “COAG” level 1 for 0.5 s to reproduce the pain that was consistent with the patient's typical experience of the original pain site (Figures 4A, B). Notably, the cycles were repeated 5 times for different neural targets as described above.

### Observation indexes

Visual analog scale (VAS): to assess pain intensity (0 points: painless, 10 points: unbearable pain) before treatment and on day 1, week 1 and month 1 after treatment.

Pittsburgh Sleep Quality Index (PSQI): to evaluate the sleep quality of patients at similar time points as VAS, with a lower score indicating higher sleep quality.

Self-Rating Anxiety Scale (SAS): to assess the anxiety level of patients. A score < 50 was considered normal, 50 to 59 was considered mild anxiety, 60 to 69 was considered moderate anxiety, and 70 or higher was considered severe anxiety (20).

Self-Rating Depression Scale (SDS): to evaluate the depression level of patients based on the SAS criteria (21).

Postoperative Satisfaction: 1 month after treatment, the satisfaction of patients with analgesic efficacy was evaluated using five levels: very satisfied (range, 80–100 points), satisfied (range, 60–79 points), average (range, 40–60 points), dissatisfied (range, 20–39 points), and very dissatisfied (0–19 point).

### Statistical analysis

Statistical analyses were performed using the SPSS software (v22.0; IBM Corporation, Armonk, NY). Quantitative data are presented as mean ± standard deviation (SD), and qualitative data are presented using frequencies. One-way analysis of variance (ANOVA) followed by LSD *t*-test or Kruskal-Wallis H-test was used to compare the multiple groups, and intragroup comparisons were performed by repeated measurement analysis of variance. Counting data were analyzed by χ^2^ test or Fisher's exact test. *P* < 0.05 indicated a statistically significant difference.

## Results

### Characteristics of patients

**Figure 5:**
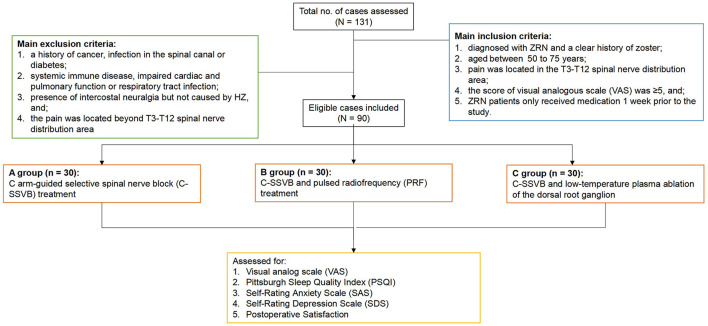
Flow chart of patient's inclusion.

A total of 131 ZRN patients were initially included in this study, and after screening by inclusion and exclusion criteria, 90 patients were finally included (Figure 5). We observed no statistically significant difference (*P* > 0.05) in the general characteristics of the three groups of patients before treatment, such as gender, age and duration of the disease (Table 1).

**Table 1:** Comparison of general information in three groups of patients.

**Parameters**	**Group A (*n* = 30)**	**Group B (*n* = 30)**	**Group C (*n* = 30)**	***F* or χ^2^ value**	***P*-value**
Age (years)	63.60 ± 5.94	65.70 ± 6.18	65.58 ± 4.18	1.38	0.258
Sex (male/female)				1.87	0.393
Male	18 (60.0%)	14 (46.7%)	13 (43.3%)		
Female	12 (40.0%)	16 (53.3%)	17 (56.7%)		
Duration of disease (days)	52.10 ± 9.86	51.46 ± 6.46	55.46 ± 7.12	2.19	0.118

### VAS scores before and after treatment in different groups

Table 2 shows no significant difference in VAS scores among the three groups before treatment and all patients in the severe category before treatment. After treatment, no significant difference was also found in VAS scores between group A and group B at different time points, although group B showed a numerically lower VAS score than group A. Significantly greater relief in pain was observed in the three groups at 1 week after treatment compared with pretreatment (*P* < 0.01), with the pain degree of group B and C patients changing from severe to moderate at 1 week and 1 month after treatment, respectively. Comparatively, the pain intensity of patients in group C changed from severe to mild 1 month after treatment and was significantly lower than group A and B.

**Table 2:** VAS scores of patients before and after treatment (mean ± SD).

**Groups**	**Before treatment**	**1 day after treatment**	**1 week after treatment**	**1 month after treatment**
A	7.11 ± 2.98	5.34 ± 2.89	4.58 ± 2.96^**^	3.93 ± 2.13^**^
B	7.32 ± 2.31	4.89 ± 3.21^**^	4.25 ± 2.17^**^	3.79 ± 1.56^**^
C	7.21 ± 2.12	4.07 ± 1.78^**#^	3.24 ± 2.03^**^	2.12 ± 1.81^**#&^

^**^P < 0.01, compared with before treatment; ^#^*P* < 0.05, compared with group A at the same time point; ^&^*P* < 0.05, compared with group B at the same time point. SD, standard deviation.

### PSQI scores before and after treatment in different groups

Our results showed that there was no statistical difference in PSQI scores between the three groups before treatment (Table 3), but the scores significantly improved with time following the treatment (all *P* < 0.01). Further, we also observed no significant difference in PSQI score between the three groups on day 1 after treatment, while a significant difference in the PSQI score between group C and A was observed as early as 1 week after treatment. In addition, after 1 month of treatment, significantly lower PSQI scores were observed between group A and B (*P* < 0.05), and group C showed statistically significant improvement in PSQI score compared with A and B (*P* < 0.05).

**Table 3:** PSQI scores of patients before and after treatment (mean ± SD).

**Groups**	**Before treatment**	**1 day after treatment**	**1 week after treatment**	**1 month after treatment**
A	15.12 ± 3.85	9.56 ± 3.96^**^	8.10 ± 3.12^**^	8.20 ± 3.22^**^
B	14.23 ± 4.21	8.37 ± 3.11^**^	7.67 ± 2.98^**^	6.17 ± 2.45^**#^
C	14.59 ± 3.51	8.19 ± 2.59^**^	5.76 ± 2.97^**#^	4.45 ± 2.77^**#&^

^**^P < 0.01, compared with before treatment; ^#^*P* < 0.05, compared with group A at the same time point; ^&^*P* < 0.05, compared with group B at the same time point. SD, standard deviation.

### Anxiety and depression in PHN patients

As can be seen in Tables 4, 5, mild anxiety and depression were both observed in PHN patients before treatment. All patients showed improvement in anxiety and depression status from 1 day to 1 month after treatment compared with pretreatment (*P* < 0.01), and the improvement tended to be more evident in group C compared with group A and B.

**Table 4:** SAS scores of patients before and after treatment (mean ± SD).

**Groups**	**Before treatment**	**1 day after treatment**	**1 week after treatment**	**1 month after treatment**
A	50.23 ± 5.25	45.14 ± 4.89^**^	37.58 ± 6.96^**^	35.93 ± 8.13^**^
B	50.92 ± 4.31	40.31 ± 5.11^**Δ^	35.45 ± 4.67^**^	30.88 ± 7.56^**Δ^
C	48.21 ± 4.82	39.27 ± 5.25^**Δ^	31.24 ± 6.87^**Δ&^	24.91 ± 4.81^**Δ*&&*^

^**^P < 0.01, compared with before treatment; ^Δ^*P* < 0.01, compared with group A at the same time point; ^&^*P* < 0.05, ^&&^*P* < 0.01 compared with group B at the same time point. SD, standard deviation.

**Table 5:** SDS scores of patients before and after treatment (mean ± SD).

**Groups**	**Before treatment**	**1 day after treatment**	**1 week after treatment**	**1 month after treatment**
A	48.53 ± 4.25	30.14 ± 4.58^**^	29.58 ± 3.96^**^	28.23 ± 2.13^**^
B	49.72 ± 5.31	30.31 ± 6.11^**^	27.45 ± 5.67^**^	28.88 ± 4.56^**^
C	50.28 ± 3.82	29.27 ± 4.38^**^	27.64 ± 4.03^**^	25.81 ± 2.41^**^

^**^P < 0.01, compared with before treatment. SD, standard deviation.

### Adverse events

One month after treatment, the adverse events of patients were assessed. As can be seen from Table 6, the incidence of adverse events in the three groups was not significantly different, with the most predominant of them being puncture pain at the surgical site and skin numbness. No serious adverse reactions occurred in any of the three groups.

**Table 6:** Comparison of treatment-related adverse events 1 month after treatment [*n* (%)].

**Groups**	**Puncture pain**	**Skin numbness**	**New onset neuralgia**	**Wound infections**	**Total**	***P*-value**
A	3 (10.0)	2 (6.7)	1 (3.3)	0 (0.0)	6 (20.0)	>0.05
B	2 (6.7)	3 (10.0)	1 (3.3)	0 (0.0)	6 (20.0)	
C	3 (10.0)	4 (13.3)	0 (0.0)	0 (0.0)	7 (23.3)	

Fisher χ^2^ test was used to compare between groups.

### Analgesics usage and patient satisfaction

After the treatment, a one-month follow-up on medication usage rate (PGB) and patient satisfaction was performed. As seen from Table 7, the medication-usage rates (PGB) in the three groups presented a significant difference (*P* < 0.01), and patients in group C showed the lowest usage rates (33.3%). Further, 1 month after treatment, the results demonstrated that the analgesic effect in group C was the highest (indicated as satisfaction scores), compared with that in group A and B (*P* < 0.05).

**Table 7:** The number of patients using analgesics and patient satisfaction after treatment.

**Index**	**Group A (*N* = 30)**	**Group B (*N* = 30)**	**Group C (*N* = 30)**	**χ^2^ or H test**	***P*-value**
Medication-usage rates (PGB)	4 (13.3)	5 (16.7)	4 (13.3)	0.180	>0.05
Analgesia satisfaction scores	49.1 ± 13.6	69.1 ± 13.6	95.0 ± 8.6	0.020	0.021

χ^2^ test was used to compare medication-usage rates; Kruskal-Wallis *H*-test was used to compare satisfaction scores.

## Discussion

ZRN is a common clinical neuropathic pain disorder usually caused by VZV infection (22), but its pathogenesis still remains unclear. Older individuals between the ages of 50 and 70 with weakened immune systems are most commonly affected by HZ (23). In China and other developed countries, herpetic neuralgia and hospitalization rates show a yearly increase with the progress of aging (24). Currently, the effective treatment method for ZRN patients remains challenging in the field of pain. Medications or anti-virus treatments are effective in PHN patients with excellent early outcomes. Nevertheless, treatment outcomes are poor for those with refractory and relapsed disease, and the patients will be at high risk of adverse events (25, 26). Several study investigated the use of physiotherapy for ZRN patients, such as nerve block or pulsed radiofrequency (27, 28). The two treatments were found to effectively lessen pain in patients with neuropathic pain and shorten recovery time (27, 29). However, each of them has its own disadvantage. Therefore, we compared the clinical efficacies and outcomes of these three therapeutic methods for ZRN patients. Our results showed that the pain intensity of ZRN patients significantly decreased under the three treatments, which was consistent with previous reports (29, 30); the pain degree of group B and C patients changed from severe to moderate at 1 month and 1 week after treatment, respectively. Besides, the VAS scores were lower in group B and C compared with group A, which indicated that the combination treatments had higher efficacy in attenuating ZRN.

In the past, several studies primarily focused on combining physical therapy and pharmacological interventions to treat ZRN or PHN patients (31, 32). Our results suggested that the combination of two physical therapy similarly had a more satisfying effect and that all the three methods had positive effects, which may be attributed to the contribution of DRG to the occurrence and development of neuropathic pain (33, 34). Studies demonstrated that during the thoracic spinal nerve block treatment, the drug diffused through the intervertebral foramen directly acted on the DRG and then effectively blocked the pain conduction of the anterior, posterior and meningeal branches of the spinal nerve (35). Under the guidance of ultrasound or C-arm, the thoracic vertebra and other tissues were visible, which was useful to guide the location of the puncture needle to the target point and prevent damage to blood vessels, nerves, etc. in the operation area. The effects directly acted on the DRG was faster than oral pain medication. LTPRA is frequently used in discogenic back pain and head and neck surgery because it can address the shortcoming of the other two methods with minimally invasive and greater safety (36, 37). Especially, our current findings revealed that only LTPRA treatment changed the degree of pain from severe to mild 1 month after treatment and showed significant improvement 1 day and 1 month after treatment.

Some patients may experience anxiety, depression, and other emotional disorders as a result of severe facial or other cutaneous nerve segment pain, which has a negative impact on their physical health, psychological health, and quality of life (38, 39). The decreasing SDS and SAS scores in our data revealed that depression and anxiety levels were significantly improved after treatment compared to the baseline. These findings indicated the significantly improved overall status of the patients after treatment. Concretely speaking, the sleep quality of patients was improved in these groups, as well as depression and anxiety with a same tendency. Notably, the reduction in bodily pain was most obvious in group C with a more pronounced score of emotional status in ZRN patients.

It was worth noting that some patients in all groups required oral analgesics on the postoperative day, which suggested the effect of the intervention may not have had the desired impact in some patients with severe pain. This was in agreement with our observation that not all groups demonstrated a clear reduction in pain (no data showed). Besides, studies have shown that medication in accordance with several therapeutic standards does not always result in the intended pain alleviation in patients with severe pain (40, 41), and conversely, numerous related adverse reactions may gradually emerge. Our findings also indicated that certain adverse events-primarily headaches and dizziness-occurred throughout the 1-month follow-up. However, it was not clearly attributed to drug therapy or physical therapy.

Despite the interesting findings reported in this study, some limitations should be clarified. First, due to the retrospective nature of this study, there could have been some unavoidable bias. Second, the number of cases was rather limited; however, we managed to balance the group distributions and baseline characteristics of the patients to improve comparability and reduce comparative biases. Third, the follow-up period was short, and long-term outcomes should be investigated. Accordingly, a larger cohort using prospective clinical settings and better design studies are still needed to confirm the optimal management of ZRN patients.

## Conclusion

In conclusion, all the three methods can significantly improve pain and the overall health of ZRN patients. The combination of C-SSVB and LTPRA was most effective in relieving pain in patients relative to C-SSVB or a combination of C-SSVB and PRF. In order to provide more concrete evidence about the optimal treatment for ZRN patients, further efficacy evaluation should be conducted at a later stage with multiple centers and a larger sample size. If necessary, new methods or multi-mode combinations, such as spinal cord electric stimulation implantation, intradermal injection, drug analgesia, should be sought out.

## Data availability statement

The original contributions presented in the study are included in the article/supplementary material, further inquiries can be directed to the corresponding author.

## Ethics statement

The studies involving human participants were reviewed and approved by Shenzhen People's Hospital (Ethics No. LL-KY-2022144-01). The patients/participants provided their written informed consent to participate in this study.

## Author contributions

Z-WZ and Z-YW contributed to study design. YZ and T-YD were responsible for data collection. JZ and QW were involved in data analysis. All authors contributed to drafting and critically reviewing the manuscript and read and approved the final manuscript.
